# Editorial: Plant metabolites in drug discovery: the prism perspective between plant phylogeny, chemical composition, and medicinal efficacy, volume IV

**DOI:** 10.3389/fphar.2025.1666900

**Published:** 2025-08-20

**Authors:** Da-Cheng Hao, Richard W. Spjut, Dinghao Deng, Chun-Nian He, Ruyu Yao

**Affiliations:** ^1^ Department of Environment Science and Engineering, Biotechnology Institute, Dalian Jiaotong University, Dalian, China; ^2^ World Botanical Associates, Bakersfield, CA, United States; ^3^ Institute of Medicinal Plant Development, Chinese Academy of Medical Sciences and Peking Union Medical College, Beijing, China; ^4^ Kunming Institute of Botany, Chinese Academy of Sciences, Kunming, China

**Keywords:** pharmacophylogeny, pharmacophylomics, phytometabolite, bioactivity, ethnopharmacology

## Introduction

**FIGURE 1:**
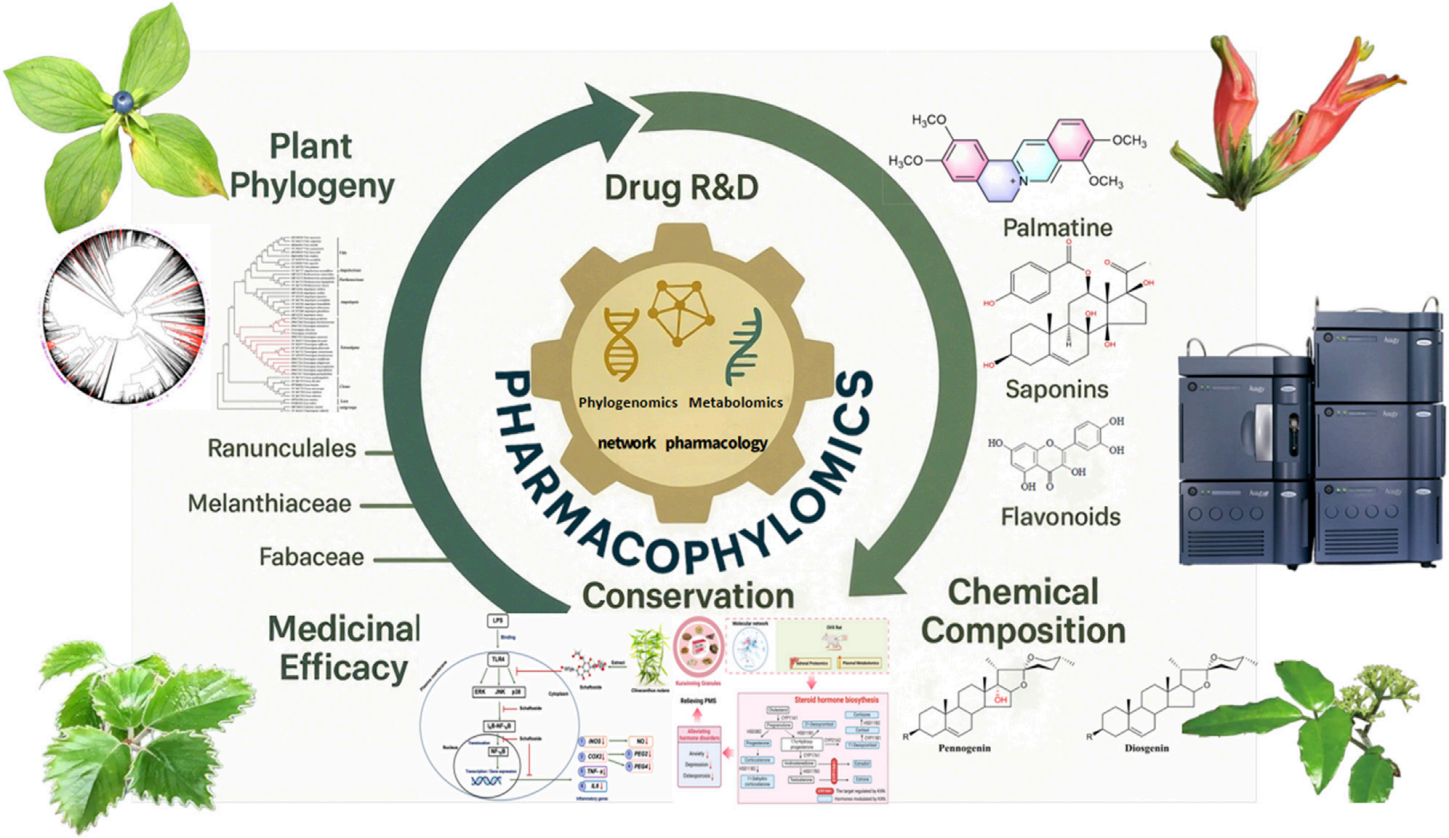
Technology driven pharmaceutical resource discovery, integrating the prism perspective of plant phylogeny, chemical composition, and pharmacology.

The intricate nexus of plant phylogeny (Chen et al., 2020; Lu and Tang, 2020), phytochemical composition, and medicinal efficacy—epitomized by the concept of pharmacophylogeny—continues to illuminate novel pathways for plant-based drug discovery (Figure 1). Building on Volumes I–III of this Research Topic (Hao et al., 2023; Hao et al., 2024a), Volume IV further unravels this prismatic relationship through cutting-edge omics technologies and interdisciplinary approaches. As phylogenetically proximate taxa often share conserved metabolic pathways and bioactivities, this framework catalyses the sustainable discovery of pharmaceutical resources amidst global biodiversity threats. The emergence of pharmacophylomics—integrating phylogenomics, transcriptomics, and metabolomics—has empowered researchers to decode biosynthetic pathways, predict therapeutic utilities, and accelerate natural product R&D (Hao et al., 2023; Hao et al., 2024a; Singh et al., 2022). This editorial synthesizes key insights from Volume IV and outlines future trajectories for the field, emphasizing how pharmacophylogeny and pharmacophylomics bridge ethnomedicine, conservation, and modern drug development.

## Volume IV highlights: pharmacophylomic integration

The eight studies in this volume span phylogenomics, metabolomics, and network pharmacology across diverse taxa, reinforcing pharmacophylogeny’s predictive power and exemplifying the power of pharmacophylomics to resolve the triad of phylogeny-chemistry-efficacy. For example, the metabolic diversity in *Paris* spp. (Melanthiaceae) is astonishing (Sun et al.); utilizing UHPLC-Q-TOF MS, researchers mapped metabolomic divergence across five newly identified *Paris* species. Terpenoids and steroidal saponins dominated chemoprofiles, with novel metabolites linked to anticancer and anti-inflammatory activities. This work underscores how phylogeny-guided metabolomics identifies taxon-specific bioactives and expands medicinal resources for phylogenetically related species. Similarly, palmatine’s multi-target ethnopharmacology is also prominent (Shi et al.). A comprehensive review highlighted palmatine—an isoquinoline alkaloid abundant in *Berberis* and *Coptis*—as a multi-target agent against inflammation, infection, and metabolic disorders. Its distribution across Ranunculales illustrates how pharmacophylogeny predicts alkaloid-rich taxa for targeted bioprospecting and validates cross-cultural ethnomedicinal uses (e.g., traditional Chinese and Ayurvedic applications). The molecular authentication of *Tetrastigma hemsleyanum* Diels et Gilg (Vitaceae) contributes to the standardized development of ethnobotanical resources (Li et al.). Chloroplast genomics and DNA barcoding resolved phylogenetic ambiguities among morphologically similar *Tetrastigma* species. The study established species-specific markers to prevent adulteration and revealed flavonoid biosynthesis genes under positive selection. This genomic groundwork ensures authentic sourcing—a critical step for pharmacology and conservation of this antipyretic TCM herb. In addition, the anti-inflammatory role of schaftoside, abundant in *C. nutans* (Burm. f.) Lindau (Acanthaceae), is also intriguing (Thongyim et al.); the metabolite profiling of Thai ethnomedicine *C. nutans* identified schaftoside (a flavone glycoside) as the primary anti-inflammatory agent in LPS-induced macrophages. Network pharmacology elucidated its synergistic regulation of NF-κB and MAPK pathways, exemplifying how phylogeny-informed metabolomics pinpoints key bioactives within complex herbal matrices.

Beyond these, pharmacophylomic approaches yielded breakthroughs in diverse therapeutic contexts. First, sphingolipidomics in *Saussureae Involucratae Herba* (Asteraceae) linked ethanol extract (SIE) to RA mitigation via modulation of SphK1/S1P signaling (Chi et al.). Second, multi-omics of Kunxinning Granules (KXN) identified astragaloside IV and icariin as CYP19A1 activators, addressing estrogen deficiency through steroid hormone biosynthesis (Wang et al.). Third, phylogenetic “hot nodes” in Fabaceae predicted phytoestrogen-rich lineages (e.g., *Glycyrrhiza*, *Glycine*) using aphrodisiac-fertility ethnomedicinal data (Thaweepanyaporn et al.). Furthermore, Kumar et al. comprehensively reviewed ethnomedicinal plants and their metabolites for snakebite therapy, identifying 116 species across 59 families. Their work exemplifies pharmacophylogeny-guided discovery: Fabaceae and Asteraceae lineages dominated antivenom taxa (39% herbs, 38% shrubs), with key phytoconstituents like terpenoids and flavonoids neutralizing venom PLA2 enzymes and hemorrhagic metalloproteinases. This underscores how phylogeny-chemistry-efficacy triangulation accelerates alternative antidote development for neglected tropical diseases.

## Pharmacophylomics: synthesis and directions

Volume IV reinforces three pillars of pharmacophylogeny and pharmacophylomics: 1. Evolution-chemodiversity links: Closely related species (e.g., *Paris* spp. or Vitaceae, palmatine in Ranunculales, Section *Glycyrrhiza* vs. *Pseudoglycyrrhiza*) share biosynthetic pathways, enabling predictive metabolite discovery. 2. Omics-driven validation: Integrating genomics, metabolomics, and network pharmacology deciphers therapeutic mechanisms and taxonomic fidelity. 3. Sustainable utilization: Phylogenomic-guided resource substitution (e.g., palmatine-rich alternatives) mitigates overharvesting threats (Hao et al., 2023; Alum, 2025). Pharmacophylomics also refines ethnopharmacology, e.g., Fabaceae “aphrodisiac-fertility hot nodes” with neurological applications (e.g., *Mimosa pudica* L., Fabaceae) showed 62% incidence of estrogenic flavonoids—validating cross-therapeutic targeting for neuro-selective phytoestrogens (Thaweepanyaporn et al.).

As a cross-cutting imperative, AI-driven predictive modeling can be highlighted; neural networks can be trained on LOTUS database (Thaweepanyaporn et al.) and phylogenomic-chemotaxonomic matrices to forecast novel bioactive lineages (e.g., neuroprotective phytoestrogens in Fabaceae). Policy integration is also imperative; Nagoya Protocol compliance can be aligned with phytochemical resource mapping, ensuring equitable benefit-sharing for indigenous knowledge holders (e.g., Thai ethnomedicines; Thongyim et al.).

## Future horizons: 3-D amalgamation and climate resilience

Future work must prioritize three synergistic dimensions to advance pharmacophylogeny and pharmacophylomics. First, horizontal expansion into uncharted taxonomic and metabolic spaces, e.g., including neglected lineages (e.g., algae, lichens) and fermentation-modified phytometabolites (Luo et al., 2024), can be a priority. For instance, the microbial-phytochemical interactions of algae and lichen symbionts (e.g., *Saussurea*-associated microbiota; Chi et al.) offer untapped biosynthetic pathways. Fermentation technologies should be scaled to transform low-yield metabolites (e.g., terpenoids in *Paris* spp.; Sun et al.) into sustainable therapeutics. In global ethnomedicinal mapping, cross-regional analyses (e.g., Fabaceae “hot nodes” in Thailand/China; Thaweepanyaporn et al.) can prioritize taxa for climate-adaptive bioprospecting. Second, in vertical integration via synthetic biology and multi-omics convergence, phylogenomics can be coupled with synthetic biology to engineer high-yield metabolites (e.g., terpenoids, alkaloids). In pathway engineering, phylogenomics-predicted biosynthetic routes (e.g., palmatine in Ranunculales; Hao et al., 2022; Shi et al.) can be leveraged to optimize high-value metabolites (e.g., CYP19A1-activated astragaloside IV in Kunxinning Granules; Wang et al.). In nano-phytocomplex delivery, targeted carriers (Kumar et al.) can be developed for venom-neutralizing phytoconstituents (e.g., terpenoid-flavonoid complexes in snakebite plants), enhancing bioavailability and reducing ecological harvest pressure. Last, in light of climate resilience through metabolic plasticity engineering, metabolomic plasticity in crops under environmental stress would be explored in depth (Hao et al., 2024b). In stress-induced chemodiversity, metabolomic shifts under abiotic stress can be characterized using proteomics and sphingolipidomics (Chi et al.). For example, *Saussurea*’s cold-adaptation mechanisms could be harnessed to engineer drought-tolerant medicinal crops. In order to improve ecophylogenetic conservation, IUCN Red List assessments can be combined with pharmacophylogenetic hot spots (e.g., *Tetrastigma* DNA-barcoded populations; Li et al.) to establish *in situ* “pharmaco-sanctuaries” for critically endangered medicinal taxa (Alum, 2025).

## Concluding remarks

As anthropogenic pressures threaten medicinal biodiversity, pharmacophylogeny/pharmacophylomics offers a robust scaffold for ethical drug discovery. Volume IV exemplifies how phylogeny and omics converge to validate ethnomedicinal knowledge—from Kunxinning’s steroid biosynthesis modulation to Fabaceae phytoestrogen prediction. We thank all contributors and encourage continued collaboration across phylogenetics, chemistry, and pharmacology. Let us harness pharmacophylomics to conserve nature’s pharmacy while advancing sustainable therapeutics. Volume IV reaffirms that the simplest truths—evolutionary kinship begets chemical kinship—remain profound guides for science.

## References

[B1] Alum E. U. (2025). Sustainable harvesting of medicinal plants: balancing therapeutic benefits with environmental conservation. Agroecol. Sustain. Food Syst. 49, 380–385. 10.1080/21683565.2024.2421948

[B2] Chen Z. D. Lu A. M. Liu B. Ye J. F. (2020). Tree of life for Chinese vascular plants. Beijing: Science Press, 1–1027.

[B3] Hao D. C. Xu L. J. Zheng Y. W. Lyu H. Y. Xiao P. G. (2022). Mining therapeutic efficacy from treasure chest of biodiversity and chemodiversity: pharmacophylogeny of Ranunculales medicinal plants. Chin. J. Integr. Med. 28 (12), 1111–1126. 10.1007/s11655-022-3576-x 35809180 PMC9282152

[B4] Hao D. C. Wang Y. X. He C. N. Spjut R. W. (2023). Editorial: plant-derived natural compounds in drug discovery: the prism perspective between plant phylogeny, chemical composition, and medicinal efficacy, volume II. Front. Plant Sci. 14, 1324514. 10.3389/fpls.2023.1324514 38023933 PMC10663306

[B5] Hao D. C. Wang Y. X. Spjut R. W. He C. N. (2024a). Editorial: plant metabolites in drug discovery: the prism perspective between plant phylogeny, chemical composition, and medicinal efficacy, volume III. Front. Pharmacol. 15, 1530039. 10.3389/fphar.2024.1530039 39734409 PMC11672199

[B6] Hao D. C. Luan Y. Wang Y. Xiao P. (2024b). Unveiling nitrogen fertilizer in medicinal plant cultivation. Agronomy 14, 1647. 10.3390/agronomy14081647

[B7] Lu A. M. Tang Y. C. (2020). The origin and evolution of primitive angiosperms. Beijing: Science Press, 1–407.

[B8] Luo X. Dong M. Liu J. Guo N. Li J. Shi Y. (2024). Fermentation: improvement of pharmacological effects and applications of botanical drugs. Front. Pharmacol. 15, 1430238. 10.3389/fphar.2024.1430238 39253373 PMC11381286

[B9] Singh K. S. van der Hooft J. J. van Wees S. C. Medema M. H. (2022). Integrative omics approaches for biosynthetic pathway discovery in plants. Nat. Prod. Rep. 39 (9), 1876–1896. 10.1039/d2np00032f 35997060 PMC9491492

